# The socio-spatial distribution of walkable environments in urban scotland: A case study from Glasgow and Edinburgh

**DOI:** 10.1016/j.ssmph.2019.100461

**Published:** 2019-07-27

**Authors:** Anna Kenyon, Jamie Pearce

**Affiliations:** University of Edinburgh, UK

**Keywords:** Spatial inequalities, Area deprivation, Walkability, Socio-spatial, Geographic information systems, Urban scotland

## Abstract

Increasingly, evidence shows that built environments (BEs) can encourage walking. Not only does walking have the potential to benefit health, it can also be used as a form of transport, reducing reliance on motorised transport and reducing CO2 emissions. However, little is known about the distribution of such features within urban environments. Furthermore, debate surrounds whether people living in areas with high deprivation face the ‘double jeopardy’ of high deprivation and environments that are unsupportive of walking.

This study aims to address this knowledge gap by developing measures of the built environment considered to support walking and assessing and whether there is a relationship between these with area-level deprivation in urban Scotland. It also examines the geographic distribution of these measures in two of Scotland's biggest conurbations. Three aspects of the physical built environment considered to reflect Area Walking Potential (AWP) were created which are considered to show good walking environments, there were residential density, intersection density and destination accessibility, as well as an overall walkability index (a combination of the three measures). The results showed no evidence of deprivation amplification with higher concentrations of the AWP measures in more deprived areas. Those living in the least deprived areas having the lowest levels of the measures. However, spatial analysis showed unequal distribution of these measures, with concentrations of high AWP clustered together with lower AWP scores in peripheral areas. These results support the growing evidence base of unequal geographic distribution of AWP. These results matter for developing built environments to support walking because it is important to understand how existing patterns of AWP to target interventions appropriately. Awareness of associations between AWP and deprivation is important for policies aimed at ameliorating multi-level inequalities demonstrating where people are likely to be experiencing both low AWP and high deprivation.

## 1 Introduction

Increasing physical activity (PA) is a pressing national and international public health concern (Department of Health, 2009, pp. 1–75; WHO, 2010). In Scotland an estimated two thirds of adults do not meet current guidelines of 150 min moderate activity per week (Physical Activity Task Force, 2003). This inactivity is associated with health risks such as increased incidence of diabetes, heart disease, worsened mental health, overweight and obesity. Moreover, there is growing evidence of inequalities in physical activity behaviour, with people experiencing higher deprivation taking part in less PA (Marmot, 2010). Interventions placing emphasis on individual-level determinants of physical activity have met with limited success (Lee, Ewing, & Sesso, 2009) and, increasingly, research is turning from a focus on the individual to the role of urban form in shaping health behaviours such as physical activity. The specific pathways through which the environment shapes health is little understood and is likely to be a complex system of interrelated influences whereby the built environment is one of many potential contributors to walking behaviours. The social ecological framework that has been used to interpret health beahviours such as walking considers behaviours to be the outcome of interactions between different types of influence operating across different levels. For example the scale at which influences operate range from individual and local to national and global. Social and physical factors have influence and individual reactions will vary according to individual characteristics and perpections (Bauman et al., 2012; Giles-Corti, Timperio, Bull, & Pikora, 2005; Saelens, Sallis, & Frank, 2003; Spence & Lee, 2003). As such the influence of the built environment is a piece of a much larger puzzle of what may influence walking. However, it is considered to be an important one with potentially far reaching consequences for a large portion of the population because neighbourhood environments provide an immediately accessible context in which many people may chose to move or walk around in. Walking is a popular form of physical activity and is considered to have the potential to increase physical activity levels across the population since it there is no cost and can be incorporated as part of daily life (The Scottish Government, 2014). It has also been identified as a potential leveller in PA participation for less active groups because it is a form of PA that most people can do and is therefore of interest to policy makers interested in increasing population level PA and reducing PA and health inequalities. As such it is a critical issue for health equity, urban and social epidemiology to understand whether neighbourhood features that are supportive of walking are distributed equitably among the population.

There is evidence that areas with diverse destinations, high street connectivity and compact residential are considered better for walking (Gunn et al., 2017), however, there is limited evidence regarding geographic distribution of these measures of AWP. Some studies have investigated patterning of urban forms, finding contrasting urban area types with increasing distance from the centre of conurbations (Siu et al., 2012, Riva, Gauvin, Apparicio, & Brodeur, 2009), indicating that urban residents have geographically unequal access to environments that are considered to support walking. This study demonstrates original evidence about the spatial patterning of these measures in urban Scotland.

It has been suggested that people may experience the ‘double jeopardy’ of higher deprivation and fewer health-supporting resources which may in turn contribute to inequalities in physical activity and related health outcomes (Shortt, Rind, Pearce, & Mitchell, 2014). This is important because there a well-documented inverse relationship between area deprivation and physical activity (Giles-Corti, 2002). Currently, evidence relating to associations between deprivation and access to AWP is mixed. Some research has suggested that area deprivation is associated with lack of physical activity resources (Estabrooks, Lee, & Gyurcsik, 2003; Taylor, Walker, Poston, Jones, & Kraft, 2006), which might discourage PA in areas with lower SES. Macintyre et al. (2007) found more community health clinics, general practices, dentists, opticians, and pharmacies in the richer compared to poorer neighbourhoods in Scotland. A recent systematic review by Jacobs et al. (2019) more positive than negative associations between walkability/bikeability and recreation resources with area deprivation but slightly more negative than positive associations between SEP and walkability/bikeability and recreational facilities with a high number of mixed and null results. The authors concluded that clear socioeconomic patterning of activity supporting environments in high income countries was not evident. A UK-based study by Zandieh, Flacke, Martinez, Jones, and van Maarseveen (2017) found this was the case for neighbourhood residential density, land-use mix, street connectivity, and retail density but found lower concentrations of greenspace and recreation facilities in more deprived neighbourhoods. Other evidence points to an equal; or even greater access to recreation and greenspace facilities in more deprived areas. King and Clarke (2015) and Gullon et al. (2017) both found positive associations between functional measures of AWP such as street connectivity and destination accessibility with area deprivation. Macintyre et al. (2007) reported greater access to recreation facilities and greenspace in more deprived areas in Scotland and Ogilvie et al. (2011) and found that the number of recreation facilities available within 10, 20 and 30 min walking and cycling thresholds in Scotland was significantly lower in the most affluent areas. Ellaway, Kirk, Macintyre, and Mutrie (2007) found that there was a higher density of children's play areas in more deprived areas of Glasgow, Scotland. This study adds to the evidence base by examining associations between AWP and area deprivation. By taking a whole country approach, using typical measures of AWP used in the literature it makes an original contribution to the current evidence base.

This study has three aims; to describe the creation of four measures of AWP, to examine associations between AWP with area deprivation and to investigate the distribution of AWP measures in conurbations surrounding Scotland's two largest cities, Edinburgh and Glasgow.

## 2 Materials and methods

### 2.1 Data

#### 2.1.1 Study sites

Urban areas of Scotland consisting of settlements of 10,000 people or more were identified using the Scottish Government's urban/rural classification (The Scottish Government, 2010). Arc GIS was used to delimit neighbourhood walking activity spaces using 1000 m network buffer zones around population weighted centroids of Scottish Output Areas (OAs) which are small administrative areas in Scotland containing approximately 50 households. 1000 m from home is considered a likely walking distance for running errands or taking a stroll for leisure and is considered appropriate for capturing a continuous walk of 10 min (McCormack, Giles-Corti, & Bulsara, 2008) required to meet national PA targets, and therefore useful for measuring walking in manner that impacts health. The resulting sample was 30,066 neighbourhoods distributed throughout urban Scotland.

#### 2.1.2 Area Walking Potential measures

Four measures of AWP were selected based on theoretical considerations and as assessment of the empirical evidence of associations with walking. These were residential density, intersection density, destination accessibility and walkability. The latter was a combination of the three former measures.

##### Residential density

Residential density measures how compact residences are across land areas. Higher density areas are considered more supportive of walking because they are likely to have greater proximity to destinations and services (Turrell, Haynes, Wilson, & Giles-Corti, 2013; Vargo, Stone, & Glanz, 2012), or may feel safer due to reduced isolation and increased observation (Forsyth, Oakes, Schmitz, & Hearst, 2007). 2001 Census data were used to count the number of addresses within each neighbourhood which was then divided by land area to calculate residential density as the number of residences per Hectare (Ha).

##### Intersection density

Intersection density is a measure of street connectivity, it can be defined as the directness and availability of alternative routes from one point to another (Handy, Boarnet, Ewing, & Killingsworth, 2002). It is hypothesised to support walking by providing direct navigation making trips shorter (Forsyth et al., 2007) and can facilitate alternative routes which may make walking more pleasant or interesting.

Data on roads and walkable trails were obtained using Ordnance Survey Integrated Transport Network dataset for roads and the Urban Paths dataset for off-road trails. These datasets were combined in Arc GIS and intersection density was calculated as the number of 3-way intersections per land area. This measure was calculated using Euclidean rather than network buffer zones around OA centroids because network buffers are created using the number of turn options so to use these to measure intersection density would be tautological. A limitation of the inclusion A roads is that some of these roads are inaccessible to pedestrians. This could result in intersection density counts over counting the turn options available to pedestrians. However, there are few turning options on such roads and therefore would result in a very limited over count of intersections. Finally, some roads are only accessible at certain times of day or may incur a fee for use but such routes are very uncommon and were not considered to substantially limit the viability of the measure.

##### Destination accessibility

Destinations may encourage walking by providing places to walk to, and in the case of open space destinations, a space in which to walk. The destinations accessibility measure was based on the National Destinations Accessibility Index (NDAI) that was developed in New Zealand (Witten, Pearce, & Day, 2011). Nine domains of destinations were used to calculate a Destinations Accessibility Index (DAI); health, public transit, education, open space, social & cultural, non-food retail, financial, food retail and employment using data from Ordnance Survey Points of Interest dataset. Neighbourhood scores were calculated based on the presence of destinations for each domain. These were summed and standardised to give a DAI score for each neighbourhood. The categories were weighted because different types of destinations are unlikely to exert an equal effect on individuals’ motivations for walking. For instance, for many people the local recreational amenities are likely to be a more regular neighbourhood destination than health service facilities and, hence, access to a range of local recreational amenities may enhance population-level physical activity more than good neighbourhood access to a General Practitioner. Therefore, a weighting, informed by theoretical rationale, the NDAI and other evidence from the literature (for example, Diez Roux et al., 2007; McCormack et al., 2008), ranging from 2 to 5 was applied to each category. Table 1 details the destination domains, subcategories, whether the subcategories were binary or tertiles and weightings.

**Table 1:** Destinations data (showing categories, subcategories data source, type weighting and rationale for inclusion) used to construct the destination accessibility index.

Primary Category	Subcategories	Data type	Possible subcategory score	weight	Weighting rationale
Health	Chemists/pharmacies	Binary	0/1	2	Occasional access but essential service
	Doctors surgeries	Binary	0/1		
Public transit	public transport stations/stops	Tertile	0–3	5	Accessed frequently, potentially used by many
Education	Secondary school	Binary	0/1	4	Accessed frequently but only by certain groups
	Primary schools	Binary	0/1		
	Pre school, afterschool	Binary	0/1		
Outdoor recreation	Accessible open space	Tertile	0–3	5	Walking destination comprising scope for walking within
Social and cultural	Sports	Binary	0/1	3	Accessed by some but not essential day to day activity
	Pubs and bars	Binary	0/1		
	Eating and drinking	Binary	0/1		
	Community centres	Binary	0/1		
	Libraries	Binary	0/1		
	Venues, stage and screen	Binary	0/1		
	Worship	Binary	0/1		
	Attractions (museums, art galleries, historical, zoological and botanical)	Binary	0/1		
Non-food retail	Clothing and accessories; household, office, leisure and garden	Tertile	0–3	4	Frequent access open to all but not as frequent as food retail
Financial	Cash machines cash points	Binary	0/1	3	Less frequent access
	Post offices	Binary	0/1		
Food retail	Supermarkets, frozen foods	Tertile	0/1	5	Access frequently and likely to be used by many
	Newsagents and tobacconists, alcoholic drinks (off-licences, wholesalers))	Binary	0/1		
	specialist shops, markets	Binary	0/1		
	convenience and general	Binary	0/1		
Employment destinations	Commercial	Tertile	0–3	2	Frequent access but only affecting those who work near to home
	Industrial	Tertile	0–3		
	Institutional	Tertile	0–3		

The open space dataset was created using data on greenspace obtained from Greenspace Scotland and beaches which was downloaded from Edina Share Geo.

##### Walkability

‘Walkability’ refers to how well an area supports walking. It has no fixed definition but is often a combined metric of several features of the BE thus reflecting overall multifaceted features of AWP. The walkability measure was constructed from the three built environment measures selected for inclusion in this study (destination accessibility, street connectivity and residential density) and so reflected multiple facets of the BE. Consideration was given to weightings used in previous measures of walkability and theoretical and empirical evidence of the relative influence of each BE measure. Intersection density was given a weighting of two which corresponds with the higher weighting given to street connectivity in other measures of walkability (Frank et al., 2006; Owen et al., 2007; Sundquist et al., 2011; Van Dyck et al., 2010) and reflects the strong associations found between street connectivity and walking in other literature (for example King & Clarke, 2015; Gullon et al., 2017). A weighting of two was applied to destination accessibility and intersection density (street connectivity), and no weight was applied to residential density. A higher weighting was applied to destination accessibility because of the strong evidence found for associations between destination accessibility and walking in the review of the literature (Cerin, Leslie, du Toit, Owen, & Frank, 2007; Glazier et al., 2014; McCormack et al., 2008; Nagel, Carlson, Bosworth, & Michael, 2008; Pikora et al., 2006; Sugiyama, Leslie, Giles-Corti, & Owen, 2009; Witten et al., 2012) Additionally, the destinations accessibility measure is based on the NDAI (Witte et al., 2011) which was designed specifically to measure destinations that are associated with walking. Residential density was given a lower ranking than the other two measures which is consistent with the approach taken in other studies (Frank et al., 2006; Owen et al., 2007; Sundquist et al., 2011; Van Dyck et al., 2010) and reflects the stronger evidence for the influence of street connectivity than for residential density in the review of the empirical literature. Furthermore, much of the evidence of positive associations between residential density and physical activity comes from a US setting where residential density is often lower than in the UK (Townshend & Lake, 2009). Finally, it was hypothesised that areas with very highest residential density scores (large high rise flats) are not necessarily conducive to walking and as such this measure was considered less important for walkability than street connectivity and destinations accessibility. Based on this reasoning, the residential density score was given a weight of 1, or not weighted.

To calculate walkability, standardised Z scores were created for each built environment measure by subtracting the mean from each data value and then dividing the result by the standard deviation using the formula:

Z =(Y_1_-Ŷ)/St. Dev

(Frank et al., 2010; Marsh & Elliot, 2008).

Walkability was calculated using the formula: 2 x z destination accessibility + z residential density + 2 x z intersection density.

#### 2.1.3 Area deprivation

The Scottish Index of Multiple Deprivation (SIMD) 2009 was used as the measure of area-level deprivation. This measure is created at datazone^1^ level for 7 measures of deprivation (Current Income, Employment, Health, Education, Skills and Training, Geographic Access to Services, Housing and Crime). A version without the Geographic Assess measures was used in this study (because destination accessibility was included as an independent variable) and each study OA was given the adapted SIMD score for the datazone into which it fell.

### 2.2 Data preparation

Neighbourhoods were ranked by scores for each of the AWP measures and area deprivation. They were then divided into quartiles where those in quartile 1 had lowest levels of the AWP measure those in and quartile 4 the highest.

## 3 Calculation

Associations between area deprivation and AWP was analysed using Spearman's rank tests of correlation, mean scores and distribution counts of neighbourhoods by quartile within deprivation quartiles. Scotland's two largest conurbations, Glasgow and Edinburgh, were selected for further spatial analysis. These areas were selected because they contain the highest number of output areas in the sample and the largest proportion of the sample (47%). The extent of clustering was analysed using Getis Ord General G statistic (G(*d*)) analysis. Spatial relationships between output areas were conceptualised using inverse Euclidean distances between output areas, so that neighbouring output areas had a larger influence on the calculation than those that were far away.

## 4 Results

### 4.1 Geographic distribution of AWP measures

Table 2 shows summary statistics for the measures of AWP. Destination accessibility scores were calculated for each neighbourhood from a possible range of 0 (lowest destination accessibility) to 33 (highest destination Scores ranged from 0 to 33 in 1000 m zones with a median of 21.85 and a mean of 20.91 indicating relatively normal distribution. The number of intersections ranged from 5.73 to 633.44 per km^2^. Mean scores were higher than the median scores for both neighbourhood zones (159.24 and 138.85 respectively) showing more areas had high than low intersection density scores showing more negative than positive scores, showing a slightly positive skew. Residential density scores ranged from 0 to 96.97 dwellings per hectare in 100 m zones. As with street connectivity the mean scores are higher than the median scores for both zones indicating that more neighbourhoods had higher residential densities than lower residential density. Standardised walkability scores had very similar mean and medians.

**Table 2:** Summary of AWP scores (n = 30,066).

BE measure	Minimum	Maximum	Mean	Median
Destination accessibility (per output area)	0.00	33.00	20.91	21.85
Residential density (per hectare)	0.00	96.97	23.36	19.75
Intersection density (per km^2^)	5.73	633.44	159.24	138.85
Walkability (per output area)	−10.15	15.29	0.00	0.26

**Fig. 1:**
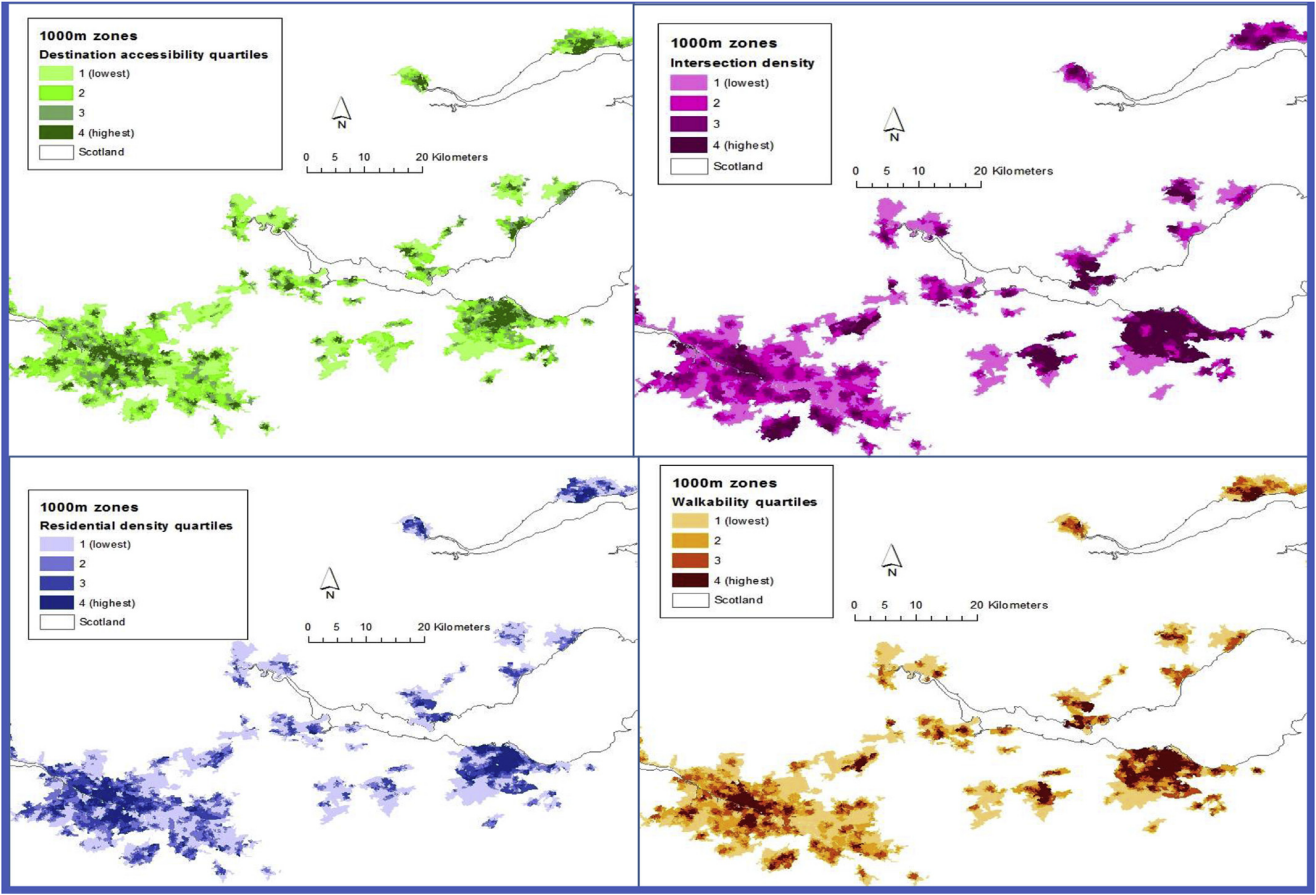
Geographical distribution of AWP measure quartiles for neighbourhoods across the central belt of Scotland.

Fig. 1 shows choropleth maps of destination accessibility, intersection density, residential density and walkability respectively across the central belt of Scotland which contained the most study sites. There was observable clustering of neighbourhoods with similar levels of the AWP measures, with concentrations of neighbourhoods with high levels of AWP surrounded by neighbourhoods with lower AWP.

Table 3 shows positive Z scores for all measures in Glasgow and Edinburgh. High positive Z scores suggest clustering of high values or a hot spot, while a cluster of high negative Z scores shows a cluster of low values. All four AWP measures displayed statistically significant clustering. In Edinburgh, clustering was highest for residential density closely followed by destination accessibility. In Glasgow, the measure displaying highest clustering was also residential density, but in Glasgow there was greater clustering of intersection density than destination accessibility. In Edinburgh, the measure displaying least clustering was intersection density. In Glasgow, the measure with the lowest clustering was destination accessibility.

**Table 3:** Getis Ord General G clustering statistics for AWP in for neighbourhoods in Glasgow and Edinburgh.

City	AWP measure	Observed general G	Expected general G	Z score
Glasgow	Destination accessibility	0.000277	0.000253	48.36
	Residential density	0.000274	0.000253	51.45
	Intersection density	0.000281	0.000253	56.17
	Walkability	0.000285	0.000253	62.81
Edinburgh	Destination accessibility	0.000364	0.000317	54.18
	Residential density	0.00036	0.000317	50.21
	Intersection density	0.000328	0.000317	27.67
	Walkability	0.000341	0.000317	39.8

P < 0.01 for all results.

This shows that residential density is the least equitably distributed and people in neighbourhoods with low residential density are least likely to be proximal to areas with higher density. Conversely street connectivity and destination accessibility appear to have slightly less inequitable distribution, although all AWP measures displayed significant clustering. This analysis also shows that spatial distribution of AWP measures considered to have the potential to support walking varies between different areas and different types of area and that people are likely to live in neighbourhoods surrounded by neighbourhoods with similar levels of AWP, making access geographically unequal.

### 4.3 Deprivation and Area Walking Potential

There were weak positive relationships between deprivation and AWP indicating that more deprived areas typically also had higher AWP (Table 4). The strongest relationship was between deprivation and residential density, which had a correlation coefficient of 0.253, this is likely to reflect the higher deprivation found in larger Scottish cities (The Scottish Government, 2011; Transport Scotland, 2005). This was followed by Walkability (r_s_ = 0.194) and Destination Accessibility (r_s_ = 0.191). The relationship between intersection density and deprivation was negligible (r_s_ = 0.048). All relationships were statistically significant at the 0.01 level.

**Table 4:** Spearman's correlation coefficients (r_s_) for relationships between deprivation and AWP.

AWP measure	r_s_
Destination accessibility	0.178
Residential density	0.253
Intersection density	0.019
Walkability	0.176

- p < 0.01 for all results.

- Higher deprivation scores indicate more deprived neighbourhoods, therefore positive rs values indicate a positive relationship between the AWP measures and increasing deprivation.

- AWP scores were created using 1000 m measures around output area centroids; deprivation scores apply to entire output areas.

However, comparing the mean scores for AWP measures within deprivation quartiles showed a more nuanced picture (Table 5). Scores were lowest in areas with the lowest deprivation (i.e. the most affluent places) for all measures, except for intersection density measured where scores were slightly lower in quartile 4 (highest deprivation). This shows that in general people living in the most affluent areas had worse AWP. Mean scores for destination accessibility and residential density scores showed small incremental increases with increasing area deprivation. For destination accessibility there was an increase of over 12% measured using 1000 m zones, with mean scores of 18.25 (95% CI 18.06–18.43) in deprivation quartile 1 to 22.31 (95% CI 22.17–22.44) in quartile 4. Mean residential density scores increased by 4.9% between deprivation quartiles 1 and 4, with an increase from a mean of 20.22 (95% CI 19.93–20.51) in quartile 1 to 25.01 (95% CI 24.77–25.25) in quartile 4. Thus, there were small increases in residential density and destination accessibility as area deprivation increased. Mean intersection density scores did not show consistent relationships; scores were higher in the two middle deprivation quartiles and lower in the highest and lowest quartiles in both size zones. Walkability scores reflected the relationships observed for destination accessibility and residential density, with low mean scores in the quartile containing lowest deprivation output areas, and similarly higher scores in the other three quartiles. However, with walkability there was no consistent increase in mean scores in the higher three deprivation quartiles. Thus, walkability scores did not show consistent variation by deprivation, but scores were higher in the two highest deprivation quartiles and lower in the lowest deprivation quartile.

**Table 5:** Mean AWP measure scores within area deprivation quartiles.

Deprivation quartile	Destination accessibility		Residential density		Intersection density		Walkability	
	Mean	(95% CI)	Mean	(95% CI)	Mean	(95% CI)	Mean	(95% CI)
1 (lowest)	18.25	(18.06–18.43)	20.22	(19.93–20.51)	153.94	(151.82–156.05)	−1.08	(-1.18—0.98)
2	21.13	(20.96–21.30)	23.96	(23.62–24.30)	167.09	(164.95–169.24)	0.28	(0.19–0.38)
3	21.94	(21.79–22.09)	24.25	(23.95–24.55)	165.83	(163.71–167.95)	0.49	(0.41–0.58)
4 (highest)	22.31	(22.17–22.44)	25.01	(24.77–25.25)	150.11	148.42–151.80)	0.30	(0.24–0.37)

**Fig. 2:**
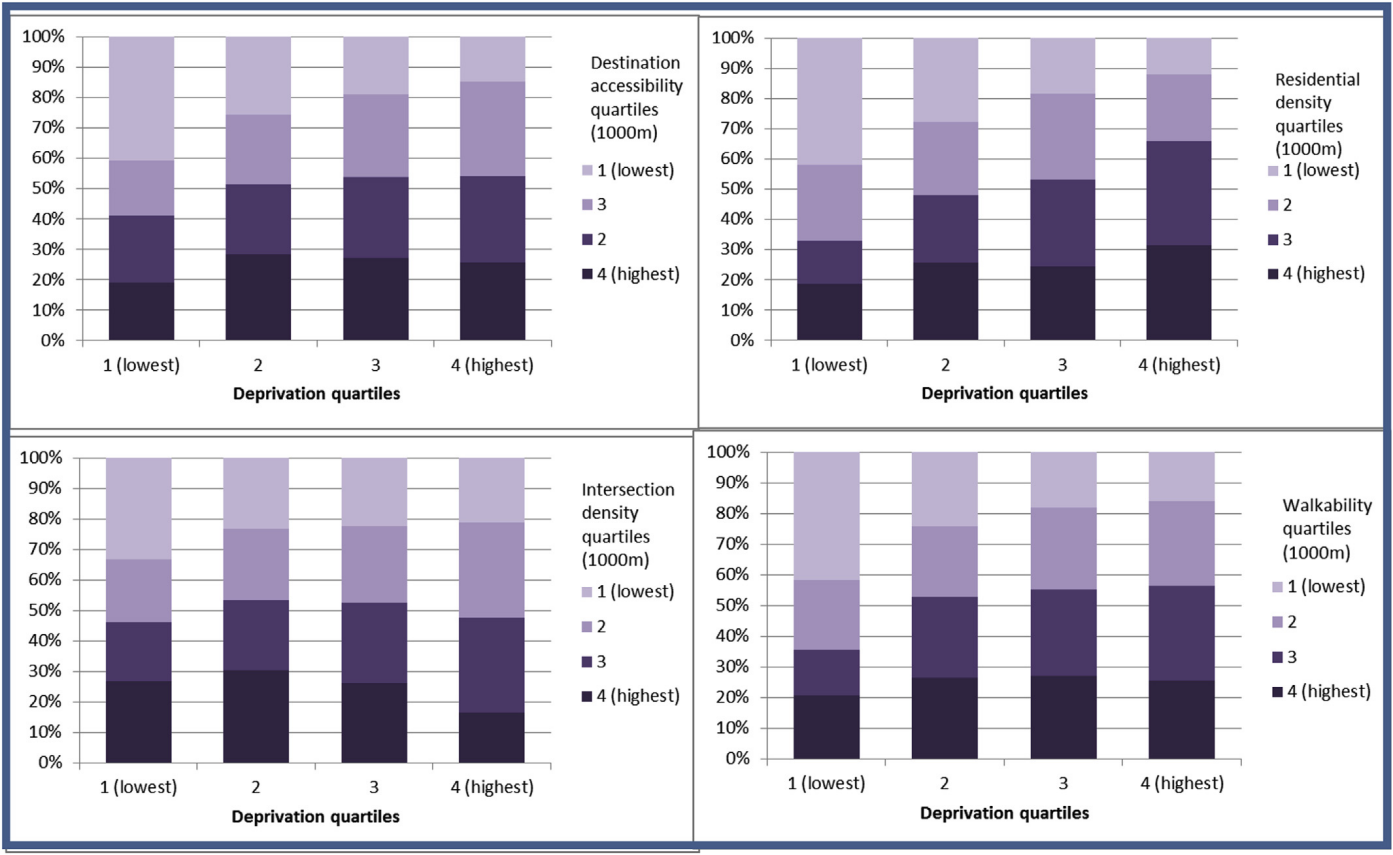
Distribution of AWP quartiles within deprivation quartiles (n = 30,066).

Fig. 2 shows the distribution of AWP measure quartiles within deprivation quartiles to show counts of each neighbourhood within deprivation quartiles. The numbers of output areas in the highest destination accessibility and residential density quartiles increased as deprivation increased, and the numbers in the lower quartiles decreased, but these trends were more marked in the lowest deprivation quartile. As deprivation increased, the number of neighbourhoods with lowest walkability decreased, however, there was little evidence of a consistent relationship between neighbourhoods with high walkability score quartiles and deprivation, the number of output areas in the highest quartiles was highest in deprivation quartiles 2 and three and lower in deprivation quartiles 1 and 4. Gamma tests of association showed that the positive relationship between deprivation quartiles and AWP was strongest for residential density with weak relationships for destination accessibility and walkability. The relationship between intersection density and deprivation was negligible (Table 6). Overall there was a consistent trend of lower AWP in areas with lower deprivation, showing that people living in the most affluent areas tended to have lower neighbourhood AWP.

**Table 6:** Gamma tests of association for correlation between deprivation and AWP quartiles (n = 30,066).

AWP measure	Gamma statistic	
	500 m zones	1000 m zones
Destination accessibility	0.195	0.180
Residential density	0.268	0.265
Intersection density	0.036	0.005
Walkability	0.198	0.178

p < 0.01 for all results.

## 5 Discussion

This study has described the creation of four measures of the built environment which can be used to explore patterns of Area Walking Potential.

The exposition of the distribution of AWP measures in Scotland's two biggest conurbations has shown that spatial distribution of AWP measures considered to have the potential to support walking is unequally distributed. There were differences in the distribution of the BE features, showing that residential density and destination accessibility were less equally dispersed than intersection density. The impact of this is that urban residents have geographically unequal access to environments that are considered to support walking depending on where they live, particularly destination accessibility and residential density. The evidence from this research is congruent with a small but growing body of evidence that has found differences in spatial patterning of AWP in relation to proximity to urban centres (Siu et al., 2012). This is likely to be because of the way in which urban areas develop, typically growing ‘outwards’ with shops and services located centrally. By contrast suburban areas were designed to provide housing, often for people who desired to be to live away from the city centre and closer to countryside with a focus on providing a spacious, quiet and safe environment rather than having high AWP (Macintyre, 2007). There were also small differences in the clustering patterns between the two conurbations which highlights that different places have different patterns of built environments, reinforcing the need for context-specific consideration of built environment interventions of AWP since patterns may vary between different countries or settlements.

Patterns of physical activity differ by area deprivation and previous work has suggested that this might be partly attributable to variations in the built environment. This study compared AWP by area deprivation and found no evidence of deprivation amplification, whereby people in more deprived areas have worse AWP in settlements in urban Scotland. Conversely, it suggested that people in the more affluent areas may have worse walking environments, and people living in areas with high deprivation do not necessarily experience low AWP. This may be to do with the historical development of urban areas, since more affluent This is congruent with other evidence from a Scottish context, finding better access to recreation facilities in areas with higher deprivation (Ogilvie, Lamb, Ferguson, & Ellaway, 2011; Ellaway et al., 2007, Macintyre et al., 2007) , although evidence from other countries finds the relationship in the opposite direction for example (Estabrooks et al., 2003; Taylor et al., 2006). This contrasts with evidence that people in more deprived areas carry out less physical activity. There are several reasons why this may be the case: It is possible that there are characteristics of higher deprivation neighbourhoods that people less likely to take part in PA. For example, there is some evidence that resources in areas with lower SES are of worse quality. Badland, Keam, Witten, and Kearns (2010) found availability of Public Open Spaces (POS) did not vary by neighbourhood deprivation in a study based in New Zealand, but found that the quality of the POS may differ by neighbourhood level SES. Jones, Hillsdon, and Coombes (2009) found the accessibility of greenspaces in England was better in more deprived areas but those residents had more negative perceptions and were less likely to use the greenspaces. This study restricted AWP measures to objective features of the BE. Forsyth (2015) notes that urban design theories make the implicit assumption that it is physical features of the built environment that will encourage walking. However, the physical environment is likely to be only a small influence on decisions about walking, which are likely to include non-physical environments such as social and policy environments as well as individual circumstances. Finally, the measures used in this study may be limited. Intersection density as measured by number of turnings has been criticised for being an oversimplified meausre that does not capture meaningful navigability (Marshall, Piatkowski, & Garrick, 2014). Additionally, safety considerations such as lack of pedestrian crossings at intersections may deter walking. Equally, areas with very highest residential density scores (large high rise flats) are not necessarily conducive to walking.

These results indicate that people living in more urban and less wealthy neighbourhoods are likely to experience high levels of the AWP measures used in this study, while their counterparts in wealthy suburban neighbourhoods have low AWP. Policy makers should be mindful of the relationship between deprivation and AWP. For patterning such as the ones found in this study, approaches to enhancing AWP that are country-wide run the risk of increasing health inequalities by enhancing environments among the least deprived sectors of the population at the expense of those in most deprived areas. Initiatives aimed at supporting walking by enhancing AWP should account for pre-existing differences in AWP in order to promote geographic equality. Using spatial analysis can help identify places where people may face, for example, both high deprivation and low AWP which can leverage spatially targeted initiatives to support people in places where there is greatest need.

## 5 Conclusion

Understanding urban form in relation to AWP enables us to understand the pattern of built environments which may influence behaviours such as walking, which may in turn influence physical activity related health outcomes. This research has described the creation of four measures of the built environment which have been used to shown that there is unequal geographic distribution of AWP in two of Scotland's largest conurbations. Information about such geographic patterns of AWP can help to target interventions appropriately and this type of information is likely to be of interest to policy makers and planners in the fields of urban design, public health and sustainability. There was no evidence that people living in areas with higher deprivation experience worse access to AWP. This adds to the growing body literature that shows that area deprivation is not correlated with walkability measured using destination accessibility, residential density and street connectivity. There is a need for further research to understand why there is an inverse relationship between deprivation and physical activity in order to tackle inequalities in participation and associated health outcomes.

## Funding

This research is based on PhD research which was funded by a grant from the Economic and Social Research Council and the Medical Research Council.

## Ethical approval

Ethical approval was obtained from the Multi-Centre Research Ethics Committee of Wales (REC reference numbers: 07/MRE09/55 and 08/MRE09/62).

## Financial interest

The authors have no affiliations with or involvement in any organization or entity with any financial interest (such as honoraria; educational grants; participation in speakers’ bureaus; membership, employment, consultancies, stock ownership, or other equity interest; and expert testimony or patent-licensing arrangements), or non-fi nancial interest (such as personal or professional relationships, affiliations, knowledge or beliefs) in the subject matter or materials discussed in this manuscript.

## Footnotes

1 Datazones are groups of contiguous output areas (OAs).
